# Early psychological intervention following the 2014 Nepal snowstorm

**DOI:** 10.1186/s40696-016-0020-9

**Published:** 2016-05-23

**Authors:** Idit Oz, Lucian Tatsa-Laur, Yitshak Kreiss, Eyal Fructer, Avraham Itzhak, Orly Sarid

**Affiliations:** 1 Israel Defense Forces, Medical Corps, Ramat Gan, Israel; 2 CIR, UCS-School of Social Work, Los Angeles, California USA; 3 Department of Military Medicine, Hebrew University, Jerusalem, Israel; 4 Department of Social Work, Ben Gurion University of the Negev, Be’er Sheva, Israel

**Keywords:** Mental health intervention, Early phase of disaster, Acute stress reaction, Acute stress disorder

## Abstract

The following is a case study of the blizzard of October 2014, an Israeli rescue team, the Special Mental Health Team (SMHT) of the Israeli Defense Forces Medical Corps, was sent to the disaster area to rescue Israeli trekkers. The SMHT intervention was provided immediately following the traumatic events with the purpose of lowering stress-related symptoms, shortening recovery time and reducing post-traumatic stress disorder symptoms that could occur in the future. Forty Israeli trekkers were assessed by SMHT: 75 % (n = 30) had mild acute stress reaction (ASR) symptoms and 25 % (n = 10) had severe acute stress disorder (ASD) symptoms. All participating trekkers receiving the intervention as a way to alleviate symptoms reported no symptoms of ASR and ASD following the intervention. Trekkers with mild ASR reported full recovery after 1 week and trekkers with ASD reported full recovery after 3 months. This case study describes the psychological intervention conducted by SMHT for the surviving trekkers following the blizzard and aims to extend the knowledge base of mental health intervention at the early phases of disaster. A research study should be conducted to develop a measurement tool capable of evaluating the effect of a short-term intervention conducted in the field.

## Background

The State of Israel considers it to be highly important to provide humanitarian assistance during disasters as well as assistance to the population during times of distress anywhere in the world. Due to these principles, the Israeli government sent a humanitarian aid delegation to Nepal following the blizzard of October 2014. As part of an Israeli effort to extend immediate medical and mental care to the survivors, rescue teams were sent to Nepal, including medical professionals and a Special Mental Health Team (SMHT) of the Israeli Defense Forces Medical Corps (IDF-MC). The SMHT has gained considerable experience in frontline early intervention missions that were conducted in war zones and in humanitarian military missions sent to natural disaster areas. These interventions were aimed at lowering the severity of symptoms occurring immediately after traumatic events as a way to shorten the recovery time [1–3] and reduce later symptoms of post-traumatic stress disorder (PTSD) [4].

The snowstorm and a series of avalanches occurred around Annapurna and Dhaulagiri, in the Manang and Mustang Districts of Nepal, within the Himalaya ridge [5]. The storm, aroused from Cyclone “Hudhud”, caused deaths of 43 people and wounded 175 persons [6–10]. Among the dead were 21 trekkers from Canada, India, Israel, Nepal, Poland, and Slovakia walking the “Annapurna Circuit” [10], as well as several local Nepali mountain guides and yak herders [11]. Injured survivors were given immediate medical attention following the disaster, and many of them suffered from ASR.

## Literature review

### Reactions to disasters

The field of mental health in disasters consists of several phases of prevention and intervention [12–15]. In the first phase, *pre*-*disaster preparation,* officials and providers planned ahead psycho-education models which included: risks and consequences to individuals, families and communities, education and mobilization of potential responders [12–15]. The second phase starts when disaster strikes*, disaster impact phase* [13, 14, 16]. Relying on Maslow’s hierarchy of needs, physical safety and security are of the utmost importance at this time. Mental reaction is often seen as a state of psychological shock, ranging from numbness and immobilization to frantic attempts to flee or heroic efforts to respond and help others [12–15]. The intervention at this stage includes psychological first aid (PFA) [12, 13, 15] such as providing information and connecting people with loved ones and resources [17].

The third phase is the *short*-*term adaptation phase*, which could last from 24 h to 3 months [12]. The loss, destruction, and grief are inescapable realities confronting individuals, families and communities. Services, community networks, and social supports are still disrupted, as are routines. During this period, mental health interventions include psychosocial identifying, validating, and normalizing reactions, and intervening with small groups and families [14].

The final phase*, recovery phase* [13], or long-term adaptation phase [12–14] is characterized by a gradual return to routine activities. Most acute phase reaction subsides by this time, yet a minority of people will develop chronic severe reactions [14].

### Intervening in disaster times

The guiding principles for early intervention are suggested in the literature aiming to promote a sense of safety and calmness, enhance self and collective efficacy, increase connectedness and hope [18]. Psychosocial recovery involves achieving these principles at the individual, group, and community levels in a manner consistent with local and cultural norms and practices [18]. Hobfoll has noted that recovery depends on individuals’ and communities’ resilience thus universal models of recovery need to be contextualized [18].

When conducting a post-disaster intervention, researchers acknowledge the importance of identifying vulnerable individuals [19]. For example, Rosenfeld [14] describes circles of vulnerability that radiate out from the disaster zone. The basic tenet is that the most vulnerable people, physically and psychosocially, are those closest to the epicenter of the disaster.

The PFA approach was developed by the National Center for Post-Traumatic Stress Disorder (NC-PTSD)—a section of the United States Department of Veterans Affairs, in 2006, in order to address the psychosocial needs of individuals in major traumatic events. Key components of this approach involves the provision of an opportunity to talk while respecting the desire not to talk about the traumatic experience; assessing basic and physical needs and ensuring that these are met; promoting positive coping mechanisms; supportive advice and linking people with sources of support; encouraging the participation in normal daily routines. Providers are called to identify those who need further help and facilitate referral to more specialist services when indicated [20].

Another model which focuses on vulnerable individuals is the critical incident stress management (CISM) model [21–24]. This model aims to normalize reactions, provide health education on possible future reactions and conduct debriefing, which is reviewing the traumatic experience, encouraging emotional expression and promoting cognitive processing [25]. For the past several years, the topic of CISM has generated a controversy in the field of disaster mental health. Practitioners and trained peer responders still use debriefings after disasters, though in an adapted form [26–31].

Previous studies on disaster intervention have focused on intervention provided to the rescue and medical teams [32–37]. Yet, very few studies have followed survivors at the short-term adaptation phase that have received a mental health intervention [38]. This case study aims to describe the psychological intervention conducted by the Israeli SMHT for the Israeli trekkers following the snowstorm disaster. Its second aim is to extend the knowledge base of mental health intervention at the early phase of disaster.

## Case presentation

### Process

The Israeli team that was sent to Nepal included search and rescue forces, volunteers, medical services and the SMHT. All worked jointly with the local Nepalese rescue team.

The SMHT arrived at Nepal 4 days following the disaster, amidst the short-term adaptation phase. The team included a psychiatrist, a social worker and a trauma surgeon who was in charge of managing the team and the provision of combined mental and physical injuries. The team stayed in Nepal for 1 week, provided assistance to whoever asked for it. However, most help seekers were Israeli trekkers.

The Israeli survivors were spread throughout the city of Katmandu. The process of locating them took about 2 days. The reaching out was made possible by teaming up with the local disaster management including officials, volunteers and rescue teams who helped to spread the word through all media and network options. Furthermore, a well-known Jewish center—”The Chabad House” in Katmandu was transformed by local officials to a support center for all of the Israeli survivors. It served as a place for therapeutic interventions.

### The trekker population

120 Israeli trekkers were trapped in the blizzard, more than 50 of them were located in the “Tharong La” mountain pass and were exposed to the most extreme blizzard conditions. Four Israelis lost their lives and seven were wounded. Out of the remaining trekkers who were in “Tharong La”, thirty trekkers independently contacted SHMT for psychological assistance and 10 trekkers were sent by local aid organizations. All in all, 40 Israeli trekkers were treated by the SMHT. All trekkers reported experiencing extreme natural conditions. They suffered from hypothermia, frost-bites, altitude sickness and snow blindness. Thirty trekkers exhibited mild ASR symptoms and 10 trekkers exhibited severe ASD symptoms.

The trekkers’ ages were between 20 and 37. The great majority were after completing their military service and few of them were college students on vacation.

### The intervention

The intervention responded to the needs of individuals in the third phase. It included PFA and CISM. The protocol was conducted as follows:

1. Reaching out—The SMHT members reached out to survivors by “actively hanging out” in the area and providing practical help as well as counseling.
2. Therapeutic alliance—were formed by the SMHT with the trekkers.
3. As recommended in the literature [39], SMHT did not replace local helping networks but rather supported and strengthened them. In practice, this meant an assessment and engagement of resourceful community personnel such as the Israeli Embassy, the ‘Chabad House’ personnel, and specific individuals involved in the disaster and rescuing efforts.
4. The SMHT identified and mapped circles of vulnerability looking for the survivors who fell into the first circle of vulnerability—the ones who had directly experienced the disaster.
5. Following the PFA [40]—vulnerable individuals were provided with physical and mental aid. Their coping skills and mental resilience were evaluated. Reactions to disaster were normalized and psycho-educational explanation about typical reactions was combined with an emphasis on self-care. Individuals were linked to sources of support, and encouraged to keep normal daily routines. The team identified those who need further help and referred them to special services when necessary.
6. Individual and group sessions were provided—following the primary assessment of trekkers by SMHT members, two groups of interventions were formed. The first group consisted of 30 participants, with mild ASR symptoms. Symptoms were assessed by the SMHT team for each individual upon joining the group and 1 week later. The group met for one session that lasted about 2 h. Elements of modified debriefing were introduced as a way of constructing a mutual narrative of the disaster.

The second group had 10 participants with severe symptoms of ASD, symptoms were assessed at three time points: T1: upon joining the group, T2: 2 days later during which they received a group intervention that lasted for 2 h and incorporated elements of modified debriefing. Participants underwent 1-3 individual sessions which focused on cognitive behavioral interventions. T3: 3 months later by phone. At T3 each individual was also assessed by answering the PTSD Checklist for DSM-5 (PCL-5) questionnaire. Group sessions were led by two SMHT members.

### Symptoms

Symptoms of ASR were assessed during an interview according to ICD-10 [41] and ASD symptoms were assessed according to the Fifth Edition of the Diagnostic and Statistical Manual of Mental Disorders [42]. Symptoms included lack of sleep, intrusive thoughts, emotional flooding, increased arousal, flashbacks, guilt and shame, levels of dissociation and emotional detachment, anxiety attacks, tantrums, and psychomotor restlessness.

PTSD symptoms in the trekkers were evaluated with the PCL-5 Questionnaire—PCL-5 Questionnaire, a 20-item self-report measure that assesses the 20 DSM-5 symptoms of PTSD [43]. Scores above 50 are defined as PTSD.

### Symptoms assessment

Seventy-five percent of the trekkers (n = 30) were assessed as having mild ASR. At the first measurement point, they reported symptoms such as crying, sadness, fear, headaches, physiological pain without injury, feelings of detachment, difficulty in concentrating and anger. At the second point, 1 week later, all trekkers reported positive improvement in their symptoms. For example, all could sleep throughout the night [44]. Trekkers commented that the group intervention assisted in normalizing their stress response and provided knowledge regarding self-care processes. They stated that building a group narrative about the disaster made them feel more resourceful and gave them a greater sense of coherence.

Twenty-five percent of the trekkers (n = 10) were assessed with severe ASD symptoms. At T1 they reported lack of sleep for more than three consecutive days, intrusive thoughts, emotional flooding, increased arousal, flashbacks of people who died in the snow, guilt and shame regarding not being able to help friends who did not survive the journey, and varying levels of dissociation and emotional detachment. They suffered from anxiety attacks, tantrums, and psychomotor restlessness. At T2 they reported a reduction in the level of their symptoms. All managed to sleep throughout the night. At T3, 3 months later, the mean score of PCL-5 scale was 19.4 ± 11.89 (median = 19.5). At T3, all ten trekkers reported no symptoms of PTSD and that they had returned to a full and healthy level of functioning in all areas of their lives. The trekkers highly valued the group intervention where they could share their cognitive, emotional and physical reactions and reflect on themselves and on other people’s range of reactions. They stated that the group discussions became part of the process of mourning and remembering. Together with the self-care strategies taught by the SMHT members, compassion and empathy to oneself and others, the trekkers managed to cope.

## Conclusions

This case study described a group intervention and elements of modified debriefing as adapted from CISM [21–23] among forty vulnerable individuals in the second phase of disaster.

All participating trekkers receiving the intervention as a way to alleviate symptoms reported no ASR and ASD symptoms following the intervention. Several explanations are offered for this outcome. The first explanation draws from the common background of the trekkers and the SMHT members. Team members and trekkers were Hebrew-speakers who served in the IDF. A common background may have contributed to the strong therapeutic alliance between the team and the trekkers. We believe that this alliance enhanced trust and support which in turn enhanced resilience. Second, during the days when the intervention took place, the SMHT maintained continuity with avalanche survivors, normalized their reactions, created cognitive restructuring and provided an ongoing sense of care, which all in all helped in lowering the level of symptoms.

It is also suggested that the group setting conducted by the SMHT helped participants in constructing a continuous narrative about what happened to them during and after the stressful situation [46]. Reduction of ASR symptoms among our participants’ corroborated results from another study showing that intervening at an early stage when memory consolidation of the traumatic event is not fully consolidated, prevents the formation of future traumatic, intrusive memories. We suggest that group intervention enhanced cohesion, decreased mental and physical isolation, and assisted in the creation of a group narrative of the event and its aftermath. This reduced ASR symptoms and enabled a better recovery [30, 45].

Ten trekkers with severe ASD symptoms, who received group and individual intervention in the early phase, fully recovered, as was measured by the PCL-5 Questionnaire 3 months later, and none developed PTSD. Another possible explanation for the decrease of symptoms is that interventions provided social support [4], which increased hope among the survivors. Hope made it possible to recruit positive mental resources and thus shaped the manner in which the traumatic events were consolidated and recalled [46, 47].

This case study shows that it would be beneficial to conduct future studies on short-term intervention after disaster and to create a measurement tool, such as a self-report questionnaire, for the evaluation of the effects of short-term intervention conducted in the field.

## Abbreviations

SMHT: Special Mental Health Team
IDF-MC: Israeli Defense Forces Medical Corps
PTSD: post-traumatic stress disorder
ASR: acute stress reaction
ASD: acute stress disorder
PFA: psychological first aid
NC-PTSD: National Center for Post-Traumatic Stress Disorder
CISM: critical incident stress management
PCL-5: PTSD Checklist for DSM-5
